# Global Regulation of Gene Expression by the MafR Protein of *Enterococcus faecalis*

**DOI:** 10.3389/fmicb.2015.01521

**Published:** 2016-01-11

**Authors:** Sofía Ruiz-Cruz, Manuel Espinosa, Oliver Goldmann, Alicia Bravo

**Affiliations:** ^1^Centro de Investigaciones Biológicas, Consejo Superior de Investigaciones CientíficasMadrid, Spain; ^2^Infection Immunology Research Group, Helmholtz Centre for Infection ResearchBraunschweig, Germany

**Keywords:** *Enterococcus faecalis*, global regulators, gene expression, carbon sources, mice peritonitis

## Abstract

*Enterococcus faecalis* is a natural inhabitant of the human gastrointestinal tract. However, as an opportunistic pathogen, it is able to colonize other host niches and cause life-threatening infections. Its adaptation to new environments involves global changes in gene expression. The EF3013 gene (here named *mafR*) of *E. faecalis* strain V583 encodes a protein (MafR, 482 residues) that has sequence similarity to global response regulators of the Mga/AtxA family. The enterococcal OG1RF genome also encodes the MafR protein (gene OG1RF_12293). In this work, we have identified the promoter of the *mafR* gene using several *in vivo* approaches. Moreover, we show that MafR influences positively the transcription of many genes on a genome-wide scale. The most significant target genes encode components of PTS-type membrane transporters, components of ABC-type membrane transporters, and proteins involved in the metabolism of carbon sources. Some of these genes were previously reported to be up-regulated during the growth of *E. faecalis* in blood and/or in human urine. Furthermore, we show that a *mafR* deletion mutant strain induces a significant lower degree of inflammation in the peritoneal cavity of mice, suggesting that enterococcal cells deficient in MafR are less virulent. Our work indicates that MafR is a global transcriptional regulator. It might facilitate the adaptation of *E. faecalis* to particular host niches and, therefore, contribute to its potential virulence.

## Introduction

The Gram-positive bacterium *Enterococcus faecalis* is usually found among the commensal microflora of the human gastrointestinal tract. However, it can become pathogenic and cause a variety of community-acquired and health care-associated infections, such as urinary tract infections, endocarditis, and bacteraemia. At present, *E. faecalis* is considered one of the most important genetic reservoirs of mobile elements, including those encoding antibiotic resistance (Fisher and Phillips, 2009; Hollenbeck and Rice, 2012). *E. faecalis* strain V583 was the first vancomycin-resistant clinical isolate reported in the United States. Its genome sequence was published in 2003 and revealed that more than a quarter of the genome consists of probable mobile or foreign DNA (Paulsen et al., 2003). *E. faecalis* strain OG1RF is a rifampicin and fusidic acid-resistant derivative of the OG1 human isolate. Its genome sequence was published in 2008 (Bourgogne et al., 2008). Compared to V583, the OG1RF genome contains 227 unique open reading frames but has fewer mobile genetic elements. Both enterococcal strains have contributed to the identification of numerous factors important for virulence (Fisher and Phillips, 2009). However, our understanding of the mechanisms involved in the pathogenicity of *E. faecalis* is still very limited. In general, global transcriptional regulators that respond to specific external signals are crucial in the adaptation of pathogenic bacteria to different host niches.

The ability to metabolize numerous carbohydrates provides enterococci with an advantage in colonizing competitive environments (Ramsey et al., 2014). The *E. faecalis* V583 genome encodes pathways for the utilization of more than 15 different sugars (Paulsen et al., 2003). Many of them are substrates for the PTS. *E. faecalis* V583 has 35 probable PTS-type sugar transporters, in addition to other transporters for sugar and polyol utilization, such as the ABC-type systems (ABC transporter family; Paulsen et al., 2003). The PTS not only transports and phosphorylates carbohydrates but also carries out regulatory functions. Some proteins have integrated a specific PTS-recognized phosphorylation domain known as the PRD (Deutscher et al., 2014). PRDs have been found in both transcriptional antiterminators and transcriptional activators that control the expression of genes involved in the uptake and metabolism of carbohydrates.

Frequently, virulence gene regulators sense changes in carbon source availability (Poncet et al., 2009), as it seems to be the case of the Mga and AtxA regulators from *Streptococcus pyogenes* and *Bacillus anthracis*, respectively. These proteins are members of an emerging class of PRD-containing global response regulators (Hondorp et al., 2013; Hammerstrom et al., 2015). Mga (530 amino acids) was shown to control the expression of ∼10% of the *S. pyogenes* genome (Ribardo and McIver, 2006). It activates the transcription of numerous genes that enable the bacterium to colonize specific host tissues and evade the host immune response (Ribardo and McIver, 2006; Hondorp and McIver, 2007). Mga is phosphorylated *in vivo* (Sanson et al., 2015) and can be phosphorylated *in vitro* by components of the PTS (Hondorp et al., 2013). AtxA (475 amino acids) was reported to have both positive and negative effects on gene expression (Bourgogne et al., 2003). It controls the expression of chromosomal genes, in addition to genes located on the virulence plasmids pXO1 (anthrax toxin genes) and pXO2 (capsule synthesis genes). Some of the AtxA-regulated genes encode secreted proteins and proteins implicated in transcriptional regulation and signaling (Bourgogne et al., 2003). The activity of AtxA is modulated by phosphorylation of histidine residues within PRDs (Tsvetanova et al., 2007). An additional member of the Mga/AtxA family of regulators is likely the Mga*Spn* protein (493 amino acids), which contributes to the virulence of *S. pneumoniae* (Hemsley et al., 2003; Solano-Collado et al., 2012, 2013). Mga, AtxA, and Mga*Spn* were predicted to have the same organization of functional domains (Hondorp and McIver, 2007). Microarray experiments showed that Mga*Spn* influences negatively the expression of several genes located within the *rlrA* pathogenicity islet (Hemsley et al., 2003). Moreover, it has been shown that Mga*Spn* activates directly the expression of a four-gene operon of unknown function (Solano-Collado et al., 2012). This regulator binds to DNA with little or no sequence specificity and no consensus DNA target was found (Solano-Collado et al., 2013).

Gene EF3013 (named *mafR* herein) of the enterococcal V583 strain encodes a 482-amino acids protein (MafR; Mga/AtxA-like *faecalis* regulator) that has sequence similarity to regulators of the Mga/AtxA family (40.7% of similarity with AtxA). Although the three-dimensional structure of this protein has been solved by X-ray crystallography (PDB 3SQN; Osipiuk et al., unpublished results), its potential role in global regulation of gene expression has not previously been investigated. In this work, we have addressed this question using genome-wide microarrays designed for strain OG1RF. We show that MafR influences positively the transcription of many genes that encode components of PTS transporters, components of ABC transporters, and proteins involved in the metabolism of carbon sources. We also show that MafR has a positive effect on the utilization of glycerol, maltose, and mannitol, suggesting that MafR may facilitate the survival of *E. faecalis* in particular host niches. Furthermore, our results suggest that a *mafR* deletion mutant strain is less virulent in a mouse peritonitis model.

## Materials and Methods

### Oligonucleotides, Bacterial Strains, and Plasmids

The oligonucleotides used are listed in **Tables 1** and **2**. The *E. faecalis* strains V583 (Paulsen et al., 2003), OG1RF (Bourgogne et al., 2008), and JH2-2 (Jacob and Hobbs, 1974) were used in this work. To construct the OG1RF Δ*mafR* mutant strain, plasmid pBVGh (thermosensitive replicon) was used (Blancato and Magni, 2010). To this end, a 560-bp region located upstream of the *mafR* gene (locus_tag OG1RF_12293) was amplified by PCR using primers 12292*Nco* and *maCla*R. Also, a 494-bp region that includes the 3′-end of *mafR* was amplified using primers *maCla*F and 12294*Nco*. Both PCR-synthesized DNAs were digested with *Cla*I, mixed in equimolecular amounts and ligated with T4 DNA ligase. The ligation mixture was then used as template for PCR amplification using primers 12292*Nco* and 12294*Nco*. The 1017-bp PCR product was digested with *Nco*I, and the restriction fragment was cloned into the *Nco*I site of plasmid pBVGh (plasmid pBVG*ΔmafR*). Strain OG1RF harboring pBVG*ΔmafR* was used to generate strain OG1RFΔ*mafR* following the protocol (integration/excision process) reported by Blancato and Magni (2010). Dye terminator sequencing at Secugen (CIB, Madrid) confirmed that OG1RFΔ*mafR* lacks the chromosomal region that spans coordinates 2421575 to 2422640. A similar procedure was used for the construction of strain JH2-2Δ*mafR*. Plasmids pAS (terminator-probe vector) and pAST (promoter-probe vector) were described (Ruiz-Cruz et al., 2010). They carry the *tetL* gene (tetracycline resistance). To construct pAST-*Pma*, a 242-bp region of the enterococcal V583 genome was amplified by PCR using the Up*Pma* and Dw*Pma* primers. The amplified DNA was digested with *Sac*I, and the 215-bp digestion product (coordinates 2888864–2889078) was inserted into the *Sac*I site of pAST. In pAST-*Pma, gfp* expression is under the control of the *Pma* promoter. To construct pAST-*Pma*Δ19 and pAS-*Pma*Δ19, a 228-bp region of the V583 genome was amplified with the Up*Pma* and Dw*Pma2* primers. After *Sac*I digestion, the 196-bp restriction fragment (coordinates 2888864–2889059) was cloned into the *Sac*I site of both pAST (pAST-*PmaΔ19*) and pAS (pAS-*PmaΔ19*). The expression vector pDLF (this work) is based on the pDL287 plasmid (LeBlanc et al., 1993), which carries a kanamycin resistance gene. pDLF has an engineered unique restriction site for *Sph*I downstream of the enterococcal *P2493* promoter (Ruiz-Cruz et al., 2010). For its construction, a 194-bp region of the V583 genome, which contains promoter *P2493*, was amplified by PCR using primers *P2493Cla*F and *P2493Cla*R. The PCR product was digested with *Cla*I, and the 171-bp restriction fragment was ligated to the *Cla*I-linearized pDL287. Plasmid pDLF*mafR* carries the *P2493*::*mafR* fusion gene. For its construction, a 1546-bp region of the OG1RF genome was amplified using the *maSph*F and *maSph*R primers. After *Sph*I digestion, the 1514-bp restriction fragment was inserted into the *Sph*I site of pDLF.

**Table 1:** Oligonucleotides used in the construction of bacterial strains and plasmids, in RT-PCR assays and in primer extension assays.

Name	Sequence (5′ to 3′)
A	GATTAGCTGAATGGTCATCGTGG
B	GTTAAAACGTGTGATAACGG
C	GACCAATCCCCTTTTTATCCG
D	AATGAAGAGTGAGCTCTGCTAG
Up*Pma*	AGGAATCAGTG**GAGCTC**CTGTCGGTAA
Dw*Pma*	GTACATGGCAA**GAGCTC**CTTTTGCTT
Dw*Pma2*	CTCCTTTTGCTTAA**GAGCTC**GGATAAAAAG
INTgfp	CATCACCATCTAATTCAACAAG
12292*Nco*	CGCAAACCTTT**CCATGG**TCAATACGACGC
*maCla*R	CCTCCTTTTGCTTAAGAAT**ATCGAT**AAAAAG
*maCla*F	CAAGAATTAATG**ATCGAT**CAAGCCGCAG
12294*Nco*	ATAACCAAACGAT**CCATGG**CGAAAGAAAG
*P2493Cla*F	AATAGGTG**ATCGAT**TGTTAAATATCTG
*P2493Cla*R	AACTCTCAC**ATCGAT**TGAACAA**GCATGC**AAAATAC
*maSph*F	TTTTTATCCGTATTC**GCATGC**AAAAGGAGG
*maSph*R	AACCAAACGAT**GCATGC**CGAAAGAAAGC

*Restriction sites are shown in bold.*

**Table 2:** Oligonucleotides used in qRT-PCR assays.

Locus tag	Forward primer (5′ to 3′)	Reverse primer (5′ to 3′)
OG1RF_10298	GGCGTTCATTACGTTGCTGA	TCGCTTCGTCAATGGTTTGAT
OG1RF_10456	AGACGCCAATTTGTTAGAACG	CACAACTAGCGGCTAAAGAAG
OG1RF_10683	CAGAAGATGGCTTACACATTACCT	GAGTAGGCTGTCCATGTCGCT
OG1RF_10684	TAATGGTCGTTGTGGCAGTA	AATTGCCCAACCGATACTCT
OG1RF_11135	CGTTCGTAGTTTTGCTGTCA	GAAGGGACAAAGCCGATTTCT
OG1RF_11146	GAGGGTGGCTTTAGTGGAGA	TTCACCTTCTGCTACGACTT
OG1RF_11592	AACAAGCCGCCTTATTTGGT	GGTTCTTCGCCAGTGTTCAT
OG1RF_11763	AACATCGGCGGTATCTTCAG	ATGCCTACATCACCAGTAGC
OG1RF_11948	CTTGTACTGATGTGACTGGGTT	CCAACGCTCCTGTAATGGTT
OG1RF_12398	CCCGACGAATGATTTGCCTA	ATCTAACGAACCAGCGACACT
OG1RF_12405	GAAGCATTGCGTTTGGAGAT	AGAAGCGACCACTTTGTTTG
OG1RF_12439	GCAACGAAATGGTGGAACAG	AAGGCATCGGCAATCTCTAAG
OG1RF_12564	ACTTGTTCGTGGACGGATTC	TGCAATGCCAACTTCTGTTA

### Growth and Transformation of Bacteria

*Enterococcus faecalis* was routinely grown in BHI medium, which was supplemented with tetracycline (4 μg/ml) when the cells harbored a plasmid based on pAS or pAST, and with kanamycin (250 μg/ml) when the plasmid was based on pDLF. Enterococcal cells containing plasmid pBVGΔ*mafR* were grown in tryptone-yeast extract broth supplemented with erythromycin (5 μg/ml). Experiments were performed at 37°C without aeration. To analyze the effect of MafR on the utilization of different carbon sources, bacteria were grown in M9 minimal medium supplemented with 0.5% yeast extract (M9YE medium) and the indicated carbon source (1% glycerol, 0.5% maltose, or 0.5% mannitol). When glycerol was used as carbon source, the medium was also supplemented with 0.26% fumarate. In these assays, 96-well microplates were used. BHI cell cultures (mid-log phase) were diluted 1:1000 in M9YE medium supplemented or not with the corresponding carbon source, and 200 μl/well was applied. Microplates were incubated at 37°C without shaking in a Thermo Scientific Varioskan Flash instrument. The bacterial growth was measured at 600 nm in 15 min cycle. Before every measurement the microplate was shaken for 10 s. The protocol used to transform *E. faecalis* by electroporation was described (Shepard and Gilmore, 1995).

### DNA Isolation

For large-scale preparations of chromosomal DNA, enterococcal cells were grown in BHI medium supplemented with 1.25% glycine. The procedure used was reported previously (Ruiz-Cruz et al., 2010). For small-scale preparations of chromosomal DNA, the Bacterial Genomic Isolation Kit (Norgen Biotek Corporation) was used. For small-scale preparations of plasmid DNA, the High Pure Plasmid Isolation Kit (Roche Applied Science) was used. The *Suspension Buffer* of this kit was supplemented with 50 mM glucose, 1.2 mg/ml lysozyme, and 240 units/ml mutanolysin. The *Lysis Buffer* (0.17 M NaOH, 1% SDS) was freshly prepared.

### Polymerase Chain Reaction (PCR)

The Phusion High-Fidelity DNA polymerase (Finnzymes) and the Phusion HF buffer were used. Reaction mixtures (50 μl) contained 5–30 ng of template DNA, 50 pmol of each primer, 200 μM each deoxynucleoside triphosphate (dNTP), and one unit of DNA polymerase. PCR conditions were reported previously (Ruiz-Cruz et al., 2010). PCR products were purified with the QIAquick PCR purification kit (Qiagen).

### RNA Isolation

For primer extension and reverse transcription-PCR (RT-PCR) assays, total RNA was isolated using the Aurum Total RNA Mini Kit (Bio-Rad). Cells were grown to an OD at 650 nm (OD_650_) of 0.5. For microarray and qRT-PCR studies, total RNA was isolated using the RNeasy mini Kit (QIAGEN). Cells were grown to an OD_650_ of 0.4. In both cases, cultures were processed as specified by the suppliers, except that cells were resuspended in buffer L (10 mM Tris-HCl, pH 8.0, 1 mM EDTA, 1 mg/ml lysozyme, 160 units/ml mutanolysin) and incubated at 37°C for 10 min. An additional DNase I digestion step was performed. The integrity of rRNAs was checked by agarose gel electrophoresis. RNA concentration was determined using a NanoDrop ND-1000 Spectrophotometer.

### Primer Extension

The ThermoScript Reverse Transcriptase enzyme (Invitrogen) and [α-^32^P]-dATP (3000 Ci/mmol; Perkin Elmer) were used. The reaction mixture (20 μl) contained 4.5 μg of total RNA and 20 pmol of primer. It was incubated at 55°C for 45 min. After heating at 85°C for 5 min, non-incorporated nucleotides were removed using Illustra MicroSpin^TM^ G-25 columns (GE Healthcare). Samples were ethanol precipitated and dissolved in loading buffer (80% formamide, 1 mM EDTA, 10 mM NaOH, 0.1% bromophenol blue, 0.1% xylene cyanol). cDNA products were analyzed by sequencing gel (8 M urea, 6% polyacrylamide). Dideoxy-mediated chain-termination sequencing reactions using DNA from M13mp18 (Yanisch-Perron et al., 1985) and primer –40 M13 (5′-GTTTTCCCAGTCACGAC-3′) were run in the same gel. Labeled products were visualized using a Fujifilm Image Analyzer FLA-3000.

### Reverse Transcription-PCR (RT-PCR)

For first-strand cDNA synthesis, 20 pmol of primer was annealed to 175 ng of total RNA. The mixture was incubated with 15 units of ThermoScript reverse transcriptase (Invitrogen) at 55°C for 45 min. PCRs were then carried out using cDNA as the template (10% of the first-strand reaction), and 20 pmol of each primer. To rule out the presence of genomic DNA in the RNA preparation, the same reactions were performed in the absence of the reverse transcriptase. As positive control, PCRs were performed with genomic DNA. PCR products were analyzed by agarose (0.8%) gel electrophoresis. Gels were stained with ethidium bromide, and DNA was visualized using a Gel-Doc system (Bio-Rad).

### Microarrays

For each strain under study, total RNA from two biological replicates was isolated. Microarray experiments were performed at Bioarray S.L. (Alicante, Spain). Specific microarrays for strain OG1RF were designed using the web-based application eArray (Agilent). The quality of the RNA preparations was assessed using a TapeStation System and the R6K ScreenTape Kit (Agilent). The Two-Color Microarray-Based Prokaryote Analysis (FairPlay III Labeling) Protocol v.1.3 from Agilent was used. Bioinformatics analysis of the microarray data was done with Bioconductor^1^ using the Limma, Marray, affy, pcaMethods, and EMA packages. The microarray data have been deposited in NCBI’s Gene Expression Omnibus (Edgar et al., 2002) and are accessible through GEO Series accession number GSE75409^2^.

### Quantitative RT-PCR (qRT-PCR)

Total RNA from three biological replicates was used. For cDNA synthesis with specific primers (validation of microarray data), the ThermoScript Reverse Transcriptase kit (Invitrogen) was used as described (see “RT-PCR” above), except that the amount of total RNA was 250 ng. In addition, the reaction mixtures contained 40 units of RNasin Plus RNase Inhibitor (Promega). Samples were twofold diluted with sterile water and stored at –80°C. For cDNA synthesis with random primers (complementation assays), the iScript Select cDNA Synthesis kit (Bio-Rad) was used. Reaction mixtures (20 μl) contained 1 μg of total RNA, 4 μl of iScript Select reaction mix, 2 μl of random primers, and 1 μl of iScript Reverse Transcriptase. Reactions were incubated at 25°C for 5 min, then at 42°C for 30 min, and finally at 85°C for 5 min. Samples were threefold diluted with sterile water and stored at –80°C. To rule out the presence of genomic DNA in the RNA preparations, reactions without adding reverse transcriptase were performed. Quantitative PCRs were carried out using the iQ SYBR Green Supermix (Bio-Rad), and a iCycler Thermal Cycler (Bio-Rad). The reaction mixtures (20 μl) contained 1–3 μl of cDNA, 10 μl of iQ SYBR Green Supermix 2x, and 500 nM of each primer. The initial denaturation step was performed at 95°C for 5 min. It was followed by 40 cycles that included the next steps: (i) denaturation at 95°C for 30 s; (ii) annealing at 55 or 60°C for 30 s; and (iii) extension at 72°C for 20 s. Data were analyzed with the iQ^TM^5 Optical System Software. Relative quantification of gene expression was performed using the comparative *C*_T_ method (Schmittgen and Livak, 2008). The internal control gene was *recA* (OG1RF_12439). The threshold cycles values (*C*_T_) of the genes of interest and the control gene were used to calculate 2^-Δ^*^C^*^T^, where Δ*C*_T_ = *C*_T_ gene of interest-*C*_T_ internal control. For a particular gene, the fold change in expression (FC) between two strains was obtained dividing the corresponding 2^-Δ^*^C^*^T^ values.

### Fluorescence Assays

JH2-2 cells carrying pAS and pAST derivatives were grown to an OD_650_ of 0.3 (logarithmic phase). Then, different volumes of the culture (25 μl to 1 ml) were centrifuged, and cells were resuspended in 200 μl of PBS buffer (10 mM Na_2_HPO_4_, 1 mM KH_2_PO_4_, 140 mM NaCl, 3 mM KCl, pH 7.2). Fluorescence intensity was measured using a Thermo Scientific Varioskan Flash instrument (excitation at 488 nm and emission at 515 nm). In each case, three independent cultures were analyzed. The fluorescence corresponding to 200 μl of PBS buffer without cells was around 0.03 arbitrary units.

### *In Silico* Prediction of Intrinsic DNA Curvature

The curvature propensity plots were calculated with the bend.it server^3^ (Vlahovicek et al., 2003). The intrinsic curvature was calculated as degrees per helical turn (10.5°/helical turn = 1°/basepair). The curvature propensity plot was calculated using the consensus scale algorithm (DNase I + nucleosome positioning data) with a windows size of 20-bp.

### Infection Model

Female BALB/c mice (8–10 weeks old) purchased from Harlan Laboratories were used for experimental infection. Mice were infected intraperitoneal with 5 × 10^7^ CFU of live *E. faecalis* and euthanized by CO_2_ inhalation at 24 h after bacterial inoculation. The infiltrating inflammatory cells were isolated from the site of infection by extensively rinsing with 2 ml of warm DMEM medium. The resulting cell suspension was counted using a Neubauer chamber and levels of inflammatory neutrophils were determined by flow cytometry analysis using anti-mouse Ly6G/C antibody (BD Pharmingen, San Diego, CA, USA). The cell suspension was also used to determine the amount of viable bacteria by plating serial dilutions on bile esculin azide agar (Fluka) plates and the levels of inflammatory cytokines. Mice were housed in a pathogen-free animal facility at the Helmholtz Centre for Infection Research and maintained under standard conditions according to institutional guidelines. Animal experiments were performed in accordance with the German regulations of the Society for Laboratory Animal Science (GVSOLAS) and the European Health Law of the Federation of Laboratory Animal Science Associations (FELASA). All experiments were approved by the ethical board Niedersächsisches Landesamt für Verbraucherschutz und Lebensmittelsicherheit, Oldenburg, Germany (permit 33.9-42502-04-12/0929).

### Cytokine Determination

The determination of IL-6 levels in the peritoneal lavage was performed by specific enzyme-linked immunosorbent assay (ELISA), using matched antibody pairs and recombinant cytokines as standards. Briefly, 96-well microtiter plates were coated with the corresponding purified anti-murine capture monoclonal anti-IL-6 (Pharmingen, San Jose, CA, USA) at a concentration of 2 μg/ml in sodium bicarbonate buffer overnight at 4°C. The wells were washed and then blocked with 1% bovine serum albumin-PBS before the serum samples and the appropriate standard were added to each well. Biotinylated rat monoclonal anti-IL-6 (BD Pharmingen) at 2 μg/ml was added as the second antibody. Detection was performed with streptavidin-peroxidase, and the plates were developed by use of TMB (3,3′,5,5′-tetramethylbiphenyl-4,4′-diamine).

### Statistical Analysis

Data were analyzed using Prism 5 (GraphPad). Statistical significance was determined by using the unpaired Student’s *t*-test for the comparison of two groups. In all analyses, *P* < 0.05 was considered statistically significant.

## Results and Discussion

### MafR is a Potential Member of the Mga/AtxA Family of Regulators

**FIGURE 1:**
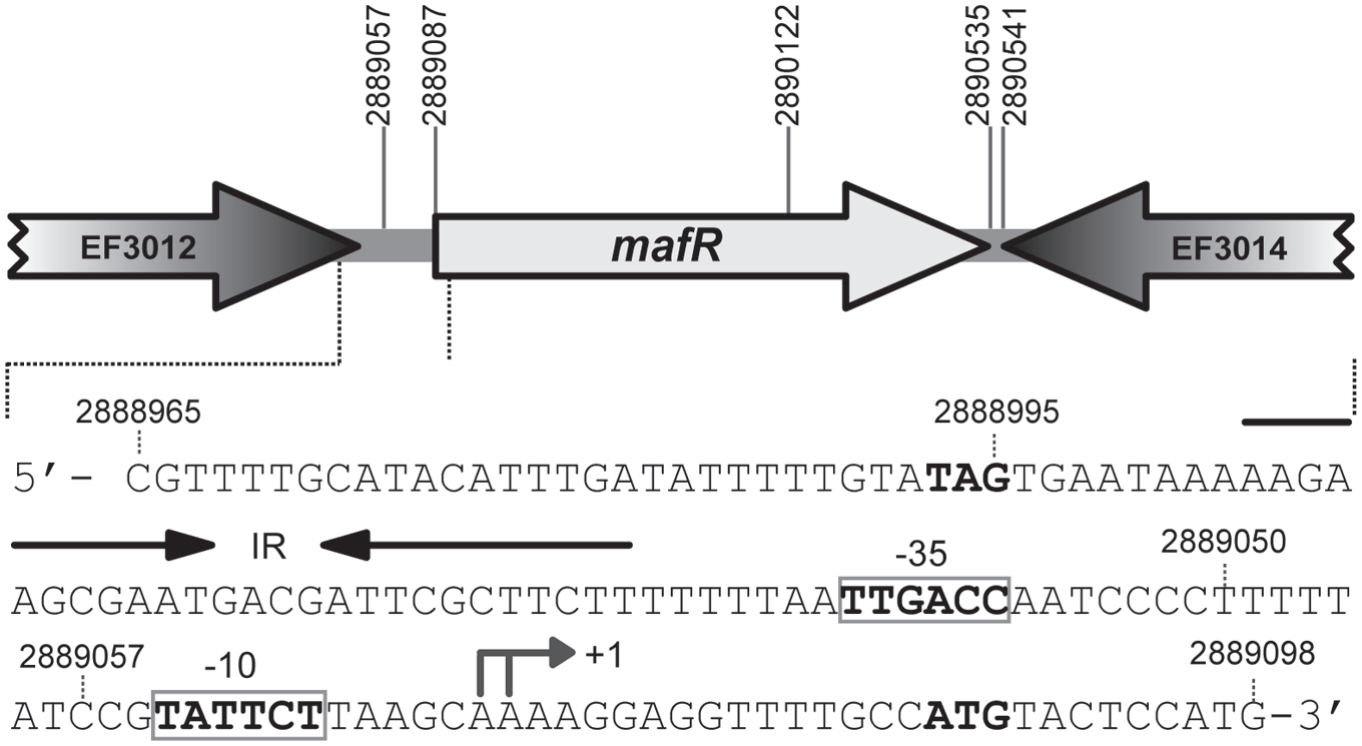
**Genetic organization of the *mafR* (locus_tag EF3013) region in the *Enterococcus faecalis* V583 genome.** The coordinates of the predicted start and stop codons are shown. The nucleotide sequence of the region spanning coordinates 2889098 to 2888965 is shown. The stop codon (TAG) of EF3012 and the start codon (ATG) of *mafR* are indicated in boldface letters. The transcription start site (+1 position) of the *mafR* gene, and the main sequence elements (–35 box and –10 box) of the *Pma* promoter identified in this work are indicated. IR: inverted-repeat.

Mga (*S. pyogenes*), AtxA (*B. anthracis*), and Mga*Spn* (*S. pneumoniae*) belong to a new family of global response regulators involved in virulence (Hondorp et al., 2013; Solano-Collado et al., 2013; Hammerstrom et al., 2015). In the genome of *E. faecalis* strain V583 (GenBank AE016830.1; Paulsen et al., 2003), the ATG codon at coordinate 2889087 is likely the translation start site of the *mafR* gene (locus_tag EF3013; **Figure 1**). It is preceded by a canonical ribosome binding site sequence (AGGAGG). Translation from this ATG codon would generate a protein of 482 residues (MafR), which is a potential transcriptional regulator of the Mga/AtxA family. According to EMBOSS needle global sequence alignment (Rice et al., 2000), MafR has 31.3/19.6, 40.7/22.1, and 38.8/23.7% of similarity/identity with Mga (530 residues; strain MGAS10394), AtxA (475 residues; strain Ames Ancestor) and Mga*Spn* (493 residues; strain R6), respectively. Moreover, in the case of MafR, analysis of its amino acid sequence using the Pfam database (Finn et al., 2008) revealed that it has two putative DNA-binding domains within the N-terminal region, the so-called HTH_Mga (Family PF08280, residues 11–69) and Mga (Family PF05043, residues 76–164) domains. Both DNA-binding domains are also present in Mga, AtxA and Mga*Spn* (Hondorp and McIver, 2007). The three-dimensional structures of MafR (EF3013; PDB 3SQN; Osipiuk et al., unpublished results) and AtxA (PDB 4R6I; Hammerstrom et al., 2015) have been solved, and it has been reported that the closest structural homolog for the AtxA C-terminal region (EIIB-like domain) is the C-terminal region of MafR (Hammerstrom et al., 2015).

### Identification of the *Pma* Promoter

**FIGURE 2:**
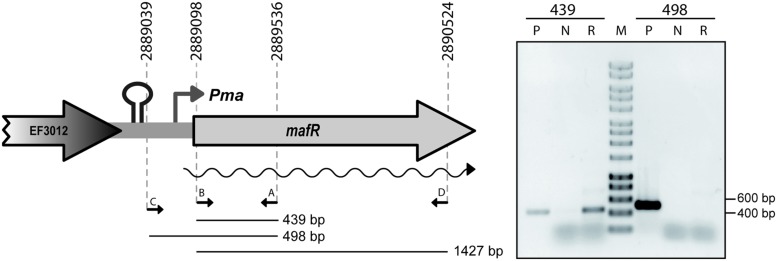
**Transcription of *mafR in vivo*.** RT-PCR assays were performed using total RNA from V583 cells. The position of the four primers (A–D) used in the experiment is shown. RT-PCRs samples (lanes R) were analyzed by agarose (0.8%) gel electrophoresis. The sizes (in bp) of the DNA regions amplified by PCR using chromosomal DNA and the corresponding pair of primers (lanes P; positive control) are indicated. RT-PCRs without adding the reverse transcriptase were also performed (lanes N; negative control). The sizes (in bp) of DNA fragments (lane M) used as molecular weight markers (HyperLadder I, Bioline) are indicated on the right of the gel.

**FIGURE 3:**
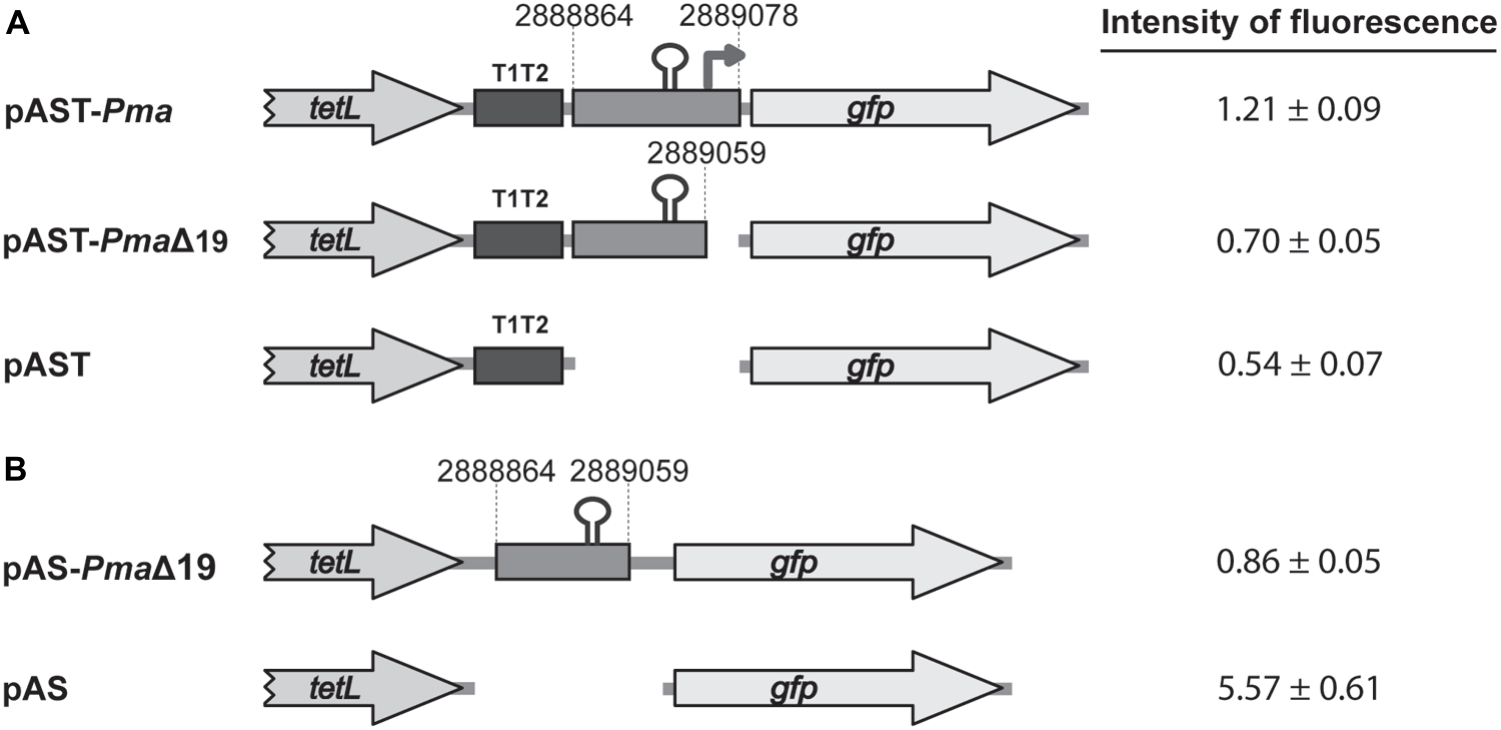
**Fluorescence assays. (A)** Activity of the *Pma* promoter. The promoter-probe vector pAST was described (Ruiz-Cruz et al., 2010). Genes *tetL* (tetracycline resistance determinant) and *gfp* (green fluorescent protein) are indicated. The T1T2 box represents the tandem terminators T1 and T2 of the *Escherichia coli rrnB* rRNA operon. The coordinates of the V583 DNA regions are indicated. The stem-loop structure represents the inverted-repeat (IR) identified upstream of the *Pma* promoter (this work). **(B)** Role of the IR element. The terminator-probe vector pAS was described (Ruiz-Cruz et al., 2010). The intensity of fluorescence (arbitrary units) corresponds to 0.8 ml of culture (OD_650_= 0.3). In each case, three independent cultures were analyzed.

**FIGURE 4:**
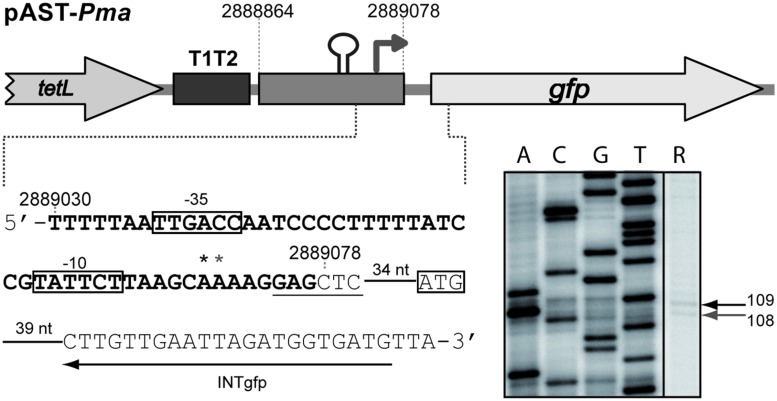
**Initiation of transcription at the *Pma* promoter.** Primer extension reactions were carried out using total RNA from JH2-2 cells harboring plasmid pAST-*Pma*. The main sequence elements of the *Pma* promoter (–35 and –10 boxes), and the ATG initiation codon of the *gfp* gene are indicated. The *Sac*I site is underlined. The asterisks indicate the 3′-end of the cDNA products synthesized using the INTgfp primer. The sizes of the cDNA products (lane R) are indicated in nucleotides on the right of the gel. Dideoxy-mediated chain-termination sequencing reactions (M13mp18 DNA and primer –40 M13) were used as DNA size markers (lanes A, C, G, T).

By RT-PCR experiments (**Figure 2**), we analyzed the expression of the *mafR* gene in enterococcal V583 cells. Oligonucleotide A was used for cDNA synthesis. The cDNA products were further amplified by PCR. With oligonucleotides A and B, a PCR product that migrated at the position expected for a 439-bp DNA was synthesized. No PCR products were detected using oligonucleotides A and C, although they were able to amplify a fragment of 498-bp when chromosomal DNA was used as template (positive control; **Figure 2**). We also performed RT-PCR assays using oligonucleotide D for cDNA synthesis. Amplification of the cDNA molecules with oligonucleotides D and B generated a product of 1427-bp (not shown). Thus, transcription of the *mafR* gene starts at a site located between coordinates 2889039 and 2889098. Next, a 215-bp region of the V583 genome (*Pma* region; coordinates 2888864 to 2889078) was inserted into the *Sac*I site of the pAST promoter-probe vector (**Figure 3A**), (Ruiz-Cruz et al., 2010), just upstream of a promoter-less *gfp* allele that encodes a variant of the green fluorescent protein. The pAST-*Pma* recombinant plasmid was then introduced into the enterococcal JH2-2 strain. Unlike strain V583 (Paulsen et al., 2003), JH2-2 is a plasmid-free strain (Jacob and Hobbs, 1974). The fluorescence in cells harboring plasmid pAST-*Pma* was 2.2-fold higher than in pAST-containing cells (**Figure 3A**), indicating that the 215-bp *Pma* region has promoter activity. Sequence analysis of such a region revealed the existence of a putative promoter (*Pma*) (**Figure 1**), which shows a 4/6 match at the –10 hexamer (5′-**TAT**TC**T**-3′) and a 5/6 match at the –35 hexamer (5′-**TTGAC**C-3′; consensus residues in promoters recognized by a σ factor similar to the *Escherichia coli* σ^70^ are shown in bold). Additional experiments confirmed that promoter *Pma* drives transcription of the *gfp* gene in plasmid pAST-*Pma*. Firstly, a deletion of 19 nucleotides that removed the –10 hexamer (*Pma*Δ19 region, coordinates 2888864–2889059) reduced the intensity of fluorescence to background levels (cells harboring plasmid pAST-*Pma*Δ19 versus cells harboring pAST; **Figure 3A**). Secondly, two cDNA extension products of 108 and 109 nucleotides were detected using total RNA from cells harboring plasmid pAST-*Pma* and the INTgfp primer (it anneals to *gfp* transcripts; **Figure 4**). Therefore, transcription of the *gfp* gene starts at the proper distance of 6–7 nucleotides from the –10 element of the *Pma* promoter.

As shown in **Figure 1**, sequence analysis of the region located between the TAG stop codon of the locus_tag EF3012 (coordinate 2888995) and the *Pma* promoter revealed the existence of an inverted-repeat followed by a short stretch of thymine residues (IR element; putative Rho-independent transcriptional terminator; **Figure 1**). To examine the efficiency of this IR element as transcriptional terminator, we inserted the *Pma*Δ19 region (196-bp; coordinates 2888864–2889059) into the pAS terminator-probe vector (**Figure 3B**), (Ruiz-Cruz et al., 2010). The recombinant plasmid pAS-*Pma*Δ19 was introduced into strain JH2-2. In pAS-carrying cells, the promoter-less *gfp* reporter gene is expressed due to the low efficiency of the *tetL* transcriptional terminator (Ruiz-Cruz et al., 2010). Compared to such cells, the fluorescence was 6.4-fold lower in cells harboring plasmid pAS-*Pma*Δ19 (**Figure 3B**). Thus, there is a transcriptional terminator signal upstream of the *Pma* promoter.

### Deletion of the *mafR* Gene in Strain OG1RF

The genome of the enterococcal OG1RF strain has been totally sequenced (GenBank CP002621.1; Bourgogne et al., 2008). The nucleotide sequence of the region that spans coordinates 2888965 to 2889098 in the V583 genome (**Figure 1**) is identical in the OG1RF genome (coordinates 2421483–2421616). By RT-PCR assays using oligonucleotides D and B (see **Figure 2**), we confirmed that the OG1RF *mafR* gene (locus_tag OG1RF_12293; coordinates 2421605–2423053) is expressed under standard laboratory conditions (BHI broth, 37°C, without aeration). Compared to strain V583, MafR of the OG1RF strain has five amino acid changes, namely A37T, Q131L, M145T, S193N, and I388S. To analyze whether MafR functions as a global transcriptional regulator, we constructed an OG1RF derivative, named OG1RFΔ*mafR*, in which the chromosomal region between coordinates 2421575 and 2422640 was deleted (coordinates 2889057 and 2890122 in the V583 genome; see **Figure 1**). This deletion removes the –10 hexamer of the *Pma* promoter and the first 344 codons of the *mafR* gene but not the IR element. Therefore, strain OG1RFΔ*mafR* is not able to synthesize MafR.

### MafR Influences Transcription of Numerous Enterococcal Genes

The OG1RF genome has 2,658 predicted genes (Bourgogne et al., 2008). By genome-wide microarrays, we obtained the transcriptional profiles of strains OG1RF and OG1RFΔ*mafR* grown to mid-log phase under standard laboratory conditions (BHI broth, 37°C, without aeration). The total number of OG1RF genes represented in the array was 2,629. In MafR-lacking cells, 90 genes were significantly differentially expressed (*P*-value <0.05): 87 genes were down-regulated (log_2_FC of mutant strain versus wild-type strain was lower than –3; see Supplementary Table S1), and three genes were up-regulated (log_2_FC of mutant strain versus wild-type strain was about 4). The latter genes were OG1RF_10454 (*fruA*, PTS family fructose porter, IIABC component), OG1RF_10455 (*fruk2*, 1-phosphofructokinase), and OG1RF_10456 (*lacR*, lactose PTS family porter repressor), which constitute an operon (*lacR-fruk2-fruA*). Thus, MafR influences negatively the transcription of such an operon. Among the 87 down-regulated genes, 15 genes encode components of PTS-type membrane transporters and nine genes encode components of ABC-type membrane transporters. In addition, and according to KEGG (Kyoto Encyclopedia of Genes and Genomes) annotations, 18 genes encode enzymes involved in carbon source metabolism (see Supplementary Table S1). In the absence of MafR, the highest reduction in gene expression (log_2_FC about –6) corresponded to the OG1RF_10296-98 operon, which is involved in mannitol utilization. A high reduction in gene expression (log_2_FC about –5) was also found in (*i*) the OG1RF_11146-49 operon, involved in glycerol metabolism, (*ii*) the OG1RF_11753 gene, which encodes the EIIBC component of a trehalose PTS transporter, and (*iii*) the OG1RF_11763-61 operon, which encodes components of a carbohydrate ABC transporter.

In some pathogenic streptococci and *Salmonella*, mutagenesis screenings identified some PTS genes as virulence factors (Turner et al., 1998; Jones et al., 2000; Hava and Camilli, 2002). Our microarray analysis showed that MafR influences positively the expression of numerous PTS genes, as *mtlA2* and *mltF2* (mannitol PTS transporter). Both genes and *mtlD* constitute the OG1RF_10296-98 operon (**Table 3**). Gene *mtlD* was shown to be significantly up-regulated during growth of *E. faecalis* in the intestinal tract of mice (Lindenstrauss et al., 2014). Among the mannose family of PTS transporters, *E. faecalis* encodes a gluconate specific EII system, which consists of four components (Zúñiga et al., 2005). It is part of a predicted metabolic pathway (gluconate utilization) that consists of two operons, OG1RF_12399-97 and OG1RF_12405-00. Both operons (nine genes) were down-regulated in the absence of MafR (log_2_FC values between –4.8 and –3.7; **Table 3**), and were shown to be up-regulated during growth of *E. faecalis* in blood (Vebø et al., 2009). Furthermore, transcription of the putative operon OG1RF_11616-11, which encodes four components of another mannose-class PTS transporter, was reduced in the absence of MafR (log_2_FC values between –4.8 and –3.3). Zúñiga et al. (2005) have suggested that mannose-class PTS transporters might play a significant role in the adaptation of bacteria to epithelial surfaces.

**Table 3:** Operons down-regulated in MafR-lacking cells.

Operon	Description	Gene validated by qRT-PCR
OG1RF_10296-98	Mannitol utilization (PTS transporter)	OG1RF_10298 (*mtlD*)
OG1RF_10683-80	Maltose metabolism	OG1RF_10683 (*malP*)
OG1RF_11003-05	ABC transporter (unknown substrate)	ND
OG1RF_11135-33	Sugar ABC transporter	OG1RF_11135
OG1RF_11146-49	Glycerol metabolism	OG1RF_11146 (*gldA*)
OG1RF_11181-88	Molybdenum cofactor biosynthesis (ABC transporter)	ND
OG1RF_11592-90	Glycerol metabolism	OG1RF_11592 (*glpK*)
OG1RF_11616-11	Mannose-class PTS transporter	ND
OG1RF_11763-61	Carbohydrate ABC transporter	OG1RF_11763
OG1RF_11951-44	Selenocompound metabolism	OG1RF_11948 (*selD*)
OG1RF_12399-97	Gluconate utilization	OG1RF_12398 (*uxuA*)
OG1RF_12405-00	(PTS transporter)	OG1RF_12405 (*gnd2*)
OG1RF_12571-60	Citrate utilization	OG1RF_12564 (*citF*)
OG1RF_12572-73		

*ND, no determined.*

MafR influences positively the expression of several ABC transporters (**Table 3**). In addition to the OG1RF_11763-61 operon (carbohydrate ABC transporter), transcription of the putative operon OG1RF_11135-33 (sugar substrate) was reduced in MafR-lacking cells (log_2_FC about –4). Expression of this operon was up-regulated during growth of *E. faecalis* in blood (Vebø et al., 2009), and was highly induced during peritoneum infection (Muller et al., 2015). Furthermore, the OG1RF_11003-05 operon (log_2_FC about –3; ABC transporter of unknown substrate) and genes OG1RF_11186-88 (molybdenum substrate) were down-regulated in the absence of MafR. The latter genes are part of an eight-gene operon (OG1RF_11181-88) that includes genes involved in molybdenum cofactor biosynthesis. Seven genes of such an operon were differentially expressed in MafR-lacking cells (log_2_FC between –2.5 and –3.7).

OG1RF_10683 (*malP*) is the first gene of a four-gene operon that includes OG1RF_10682 (*malB*), OG1RF_10681 (*malM*) and OG1RF_10680 (*malR*). The *malPBMR* operon and its neighboring OG1RF_10684 gene (*malT*, PTS transporter) are divergently transcribed and were shown to be involved in maltose utilization (Le Breton et al., 2005). The *malPBMR* operon was originally designated *bopABCD* (biofilm on plastic surfaces) because it was found to influence biofilm formation (Hufnagel et al., 2004). A mutational analysis in this operon showed a correlation between the ability of *E. faecalis* to form biofilm and its ability to colonize the mouse intestinal tract (Creti et al., 2006). The *malPBMR* operon was found to be partly up-regulated during growth of *E. faecalis* in human urine (Vebø et al., 2010). Moreover, two genes of the operon (*malP* and *malB*) and *malT* were up-regulated during growth of *E. faecalis* in blood (Vebø et al., 2009). Our microarray results revealed that *malP, malB*, and *malT* were down-regulated in MafR-lacking cells (log_2_FC about –4; **Table 3**), suggesting that MafR might play a positive role in biofilm production. Genes OG1RF_11951 (selenium-dependent molybdenum hydroxylase) and OG1RF_11948 (*selD*; selenide, water dikinase) were also down-regulated in the absence of MafR (log_2_FC about –3). Both genes, which form part of a putative eight-gene operon (OG1RF_11951-44 operon), were reported to contribute positively to biofilm formation (Ballering et al., 2009; Srivastava et al., 2011).

*Enterococcus faecalis* is able to use citrate as the sole source of carbon and energy. Citrate metabolism has been extensively investigated in this bacterium (Blancato et al., 2008; Suárez et al., 2011; Repizo et al., 2013). The *cit* locus is constituted by two divergent operons: *citHO* and *oadHDB-citCDEFX-oadA-citMG*. Our microarray analysis revealed that the *cit* locus (OG1RF_12572-73 and OG1RF_12571-60) was down-regulated in the absence of MafR (**Table 3**). With the exception of *citO* (OG1RF_12573; log_2_FC –1.66) and *citG* (OG1RF_12560; log_2_FC –2.46), the log_2_FC value of the *cit* genes was between –5.38 and –3.07 (see Supplementary Table S1). Therefore, MafR has a positive effect on the transcription of the two operons involved in citrate utilization. Several genes of the *cit* locus were up-regulated during growth of *E. faecalis* in human urine (Vebø et al., 2010). Furthermore, genes responsible for citrate utilization were found to be up-regulated during the adaptation of *E. faecalis* to blood (Vebø et al., 2009).

Glycerol can be a carbon/energy source for several pathogenic bacteria (Ramsey et al., 2014). Two pathways for glycerol catabolism are present in *E. faecalis*, named GlpO/GlpK and GldA/DhaK. The *glpK, glpO*, and *glpF* genes (OG1RF_11592-90) constitute an operon. Our microarray analysis showed that this operon was down-regulated in MafR-lacking cells (log_2_FC about –3). Also, the OG1RF_11146-49 operon, which includes genes *gldA* and *dhaK*, was down-regulated (log_2_FC about –5; **Table 3**). Hence, MafR influences positively the transcription of both glycerol catabolic operons, although the activator effect seems to be greater on the expression of the GldA/DhaK pathway. Both operons were up-regulated during the adaptation of *E. faecalis* to the intestinal tract of mice (Lindenstrauss et al., 2014), and also during mice peritonitis (Muller et al., 2015). Moreover, enterococcal mutant strains unable to metabolize glycerol were affected in organ colonization in a systemic murine infection model (Muller et al., 2015). Vebø et al. (2009) reported that the *glpKOF* operon is highly up-regulated during growth of *E. faecalis* in blood. We hypothesize that MafR may facilitate the survival of *E. faecalis* in particular host niches through the transcriptional activation of the glycerol catabolic operons.

Many eukaryotic proteins are glycosylated, including some proteins of the immune system (Apweiler et al., 1999). Proteolytic and glycolytic modulation of the host immune system appears to be a common theme in the pathogenesis of some Gram-positive bacteria (Collin and Olsén, 2003). *E. faecalis* encodes several putative glycosidases but little is known about their biological function. The endo-β-*N*-acetylglucosaminidase EndoE (OG1RF_10107) was shown to cleave the N-linked glycans of both the human immunoglobulin G (Collin and Fischetti, 2004) and the human glycoprotein lactoferrin (Garbe et al., 2014), which is considered to be a component of innate immunity. Gene OG1RF_12167 also encodes an endo-β-*N*-acetylglucosaminidase activity (Bøhle et al., 2011). Our microarray analysis revealed that transcription of both genes (OG1RF_10107 and OG1RF_12167) was highly reduced in the absence of MafR (log_2_FC about –4; see Supplementary Table S1).

### Validation of the Microarray Results by qRT-PCR

**FIGURE 5:**
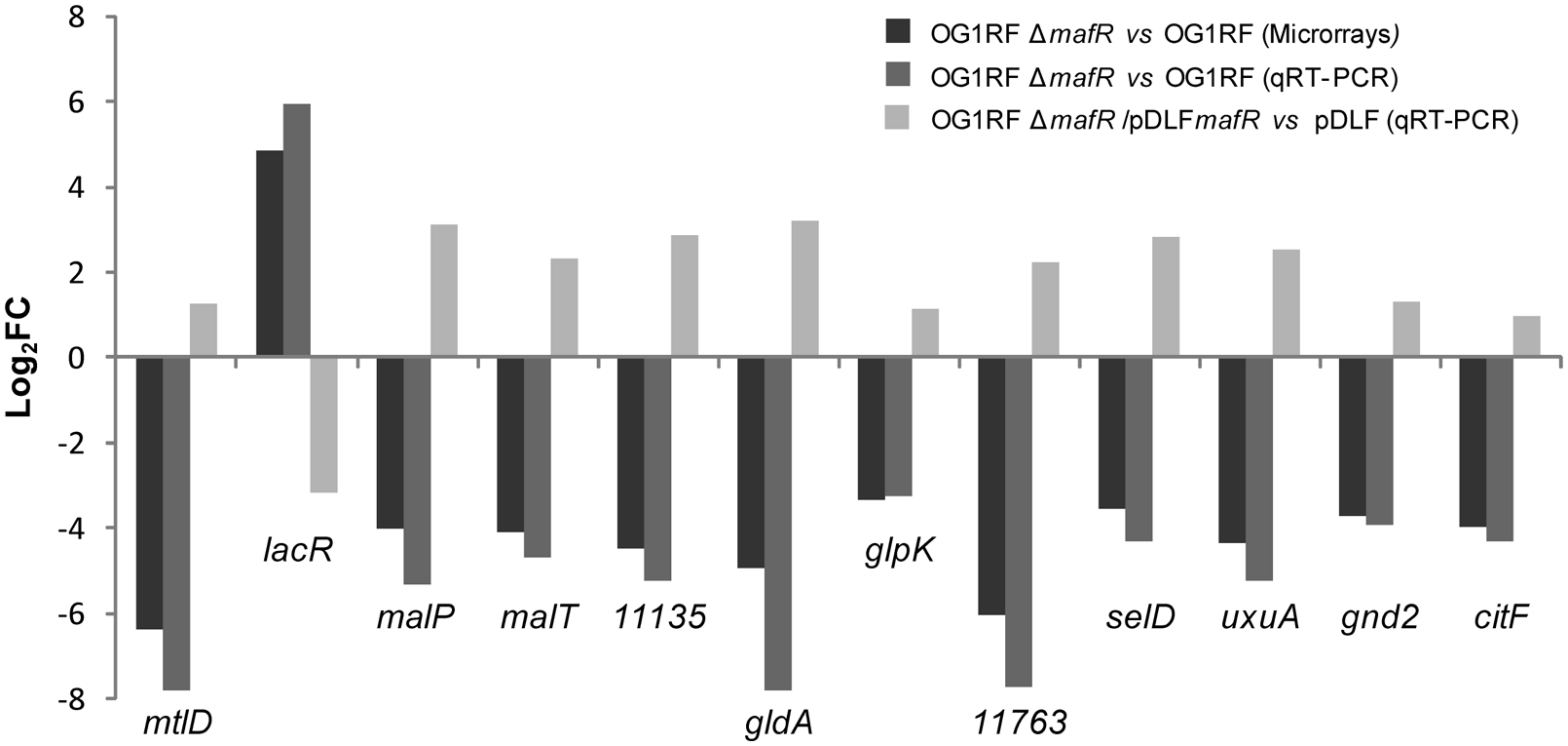
**Effect of MafR on gene expression.** Log_2_ fold change in the expression of the indicated genes due to the lack of MafR (OG1RFΔ*mafR* vs. OG1RF) determined by microarray analysis and by qRT-PCR. Log_2_ fold change in gene expression due to the presence of the MafR-encoding plasmid (OG1RFΔ*mafR*/pDLF*mafR* vs. OG1RFΔ*mafR*/pDLF).

To validate the microarray results, we performed qRT-PCR assays using the comparative *C*_T_ method (Schmittgen and Livak, 2008). Specifically, we selected 12 potential MafR target genes involved in metabolism (*mtlD, malP, gldA, glpK, selD, uxuA, gnd2, citF*), regulation (*lacR*) or transport (*malT*, OG1RF_11135, OG1RF_11763; **Table 3**). These genes are transcribed from different promoters. We determined their relative expression in OG1RF and OG1RFΔ*mafR* using *recA* as internal control gene (**Figure 5**). Except for *lacR*, gene expression was reduced in strain OG1RFΔ*mafR* compared to OG1RF. In all the cases, the fold change in gene expression (log_2_FC) due to the lack of MafR (OG1RFΔ*mafR* versus OG1RF) was comparable to the value obtained in the microarray experiments (**Figure 5**). Additionally, we performed qRT-PCR assays using total RNA isolated from strains JH2-2 and JH2-2Δ*mafR*. In this case, we determined the relative expression of genes *lacR, mtlD, gldA*, and *citF*. The results obtained confirmed that MafR has a negative effect on the transcription of *lacR* but a positive effect on the transcription of *mtlD, gldA*, and *citF* (not shown).

### Genetic Complementation Studies in Strain OG1RFΔ*mafR*

For genetic complementation of the *mafR* deletion, we inserted the *mafR* gene into the multicopy pDLF expression vector (this work), and introduced the pDLF*mafR* recombinant plasmid into the *mafR* deletion mutant strain (OG1RFΔ*mafR*). By qRT-PCR, we determined the relative expression of the 12 genes mentioned above in both strains: OG1RFΔ*mafR* harboring pDLF*mafR* (plasmid-encoded MafR) and OG1RFΔ*mafR* harboring pDLF (absence of MafR). The fold change in gene expression (log_2_FC) due to plasmid-encoded MafR is shown in **Figure 5**. Compared to cells harboring pDLF, gene *lacR* was down-regulated in cells harboring pDLF*mafR*. On the contrary, the other 11 genes were up-regulated. Hence, plasmid-encoded MafR increases the transcription of the 11 chromosomal genes. Nevertheless, such an increase is lower than the one due to the single chromosomal copy of *mafR* (see OG1RFΔ*mafR* versus OG1RF in **Figure 5**). This result suggests that only part of the plasmid-encoded MafR protein is active.

### Potential Curvatures in Promoter Regions of MafR-Regulated Operons

MafR is thought to be a member of the Mga/AtxA family of global response regulators. These regulators appear to bind DNA with low sequence specificity. Sequence alignments of all established Mga-binding regions revealed that they exhibit only 13.4% identity (Hause and McIver, 2012). In the case of AtxA, *in silico* analyses revealed that the promoter regions of several target genes are intrinsically curved (Hadjifrangiskou and Koehler, 2008). Furthermore, *in vitro* studies showed that Mga*Spn* interacts with DNA sites that contain a potential intrinsic curvature (Solano-Collado et al., 2013). Recently, Innocenti et al. (2015) mapped numerous transcription start sites on the V583 genome using a modified RNA-seq approach. Their study allowed us to locate the transcription start sites of six MafR-regulated operons on the OG1RF genome. The first gene of each operon and its transcription start site are listed in **Table 4**. We calculated the curvature propensity plots of the corresponding promoter regions (positions –1 to –200) using the bend.it program (Vlahovicek et al., 2003). Compared with the global A + T content (62.2%) of the OG1RF genome, five out of the six promoter regions display a high A + T content (74–77%). Moreover, they contain one peak of potential sequence-dependent curvature with a magnitude higher than 14 degrees per helical turn. The peak of such a curvature is located at position –171 in gene *glpK*, and between positions –63 and –86 in genes *gldA*, OG1RF_11763, *gnd2*, and OG1RF_12571 (**Table 4**). Further work is required to determine whether MafR is able to bind to DNA containing such potential intrinsic curvatures.

**Table 4:** Potential intrinsic curvatures within promoter regions.

Locus tag (gene)	TSS position^a^	A + T^b^ (%)	Curvature^c^ (°/helical turn)	Location^d^
OG1RF_10296 (*mtlA2*)	310152	64.5	12.02	–40
OG1RF_11146 (*gldA*)	1197479	75.5	13.16	–179
			16.16	–63
OG1RF_11592 (*glpK*)	1660384	77	17.35	–171
OG1RF_11763	1846229	74.5	14.64	–86
			15.01	–78
OG1RF_12405 (*gnd2*)	2542744	74	12.92	–136
			14.27	–76
OG1RF_12571	2733071	76.5	14.83	–85
			13.71	–24

*^a^Position of the transcription start site on the OG1RF genome.*

*^b^A + T content of the promoter region (from position –1 to position –200).*

*^c^Predicted curvatures with a magnitude > 12 degrees/10.5 bp helical turn.*

*^d^Location of peaks of potential curvatures within the promoter regions (relative to the TSS).*

### MafR Plays a Positive Role in the Utilization of Different Carbon Sources

**FIGURE 6:**
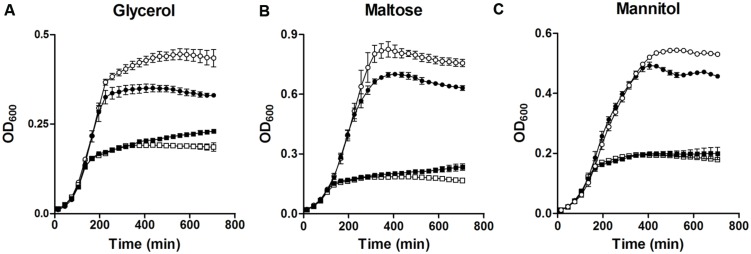
**Effect of MafR on the utilization of different carbon sources.** Bacteria were grown in M9YE medium supplemented (circles) or not (squares) with glycerol **(A)**, maltose **(B)**, or mannitol **(C)**. White symbols correspond to strain OG1RF. Black symbols correspond to strain OG1RFΔ*mafR*. Data correspond to the mean values of three independent experiments. Error bars indicate standard error of mean.

Two operons for glycerol metabolism are present in *E. faecalis*. Both of them were down-regulated in MafR-lacking cells (**Table 3**, **Figure 5**). To evaluate the effect of MafR on the utilization of glycerol, we analyzed the growth of strains OG1RF and OG1RFΔ*mafR* in M9YE medium supplemented or not with glycerol (**Figure 6A**). In the absence of glycerol, both cultures reached a similar OD_600_ (about 0.2). However, in glycerol-supplemented medium, the final OD_600_ reached in cultures of the mutant strain was lower than that attained in cultures of the wild-type strain. Furthermore, compared to the wild-type strain, the MafR-lacking strain showed a diminished growth in M9YE medium supplemented with maltose (**Figure 6B**), which correlates with the lower expression found in genes *malP, malB*, and *malT* (maltose utilization; **Table 3**, **Figure 5**). The growth of the mutant strain was also reduced in M9YE medium supplemented with mannitol (**Figure 6C**), which is linked to the reduction in expression of the OG1RF_10296-98 operon (mannitol utilization; **Table 3**, **Figure 5**). Therefore, MafR has a positive effect on the utilization of glycerol, maltose, and mannitol.

### MafR Influences the Host Inflammatory Response During Mice Peritonitis

**FIGURE 7:**
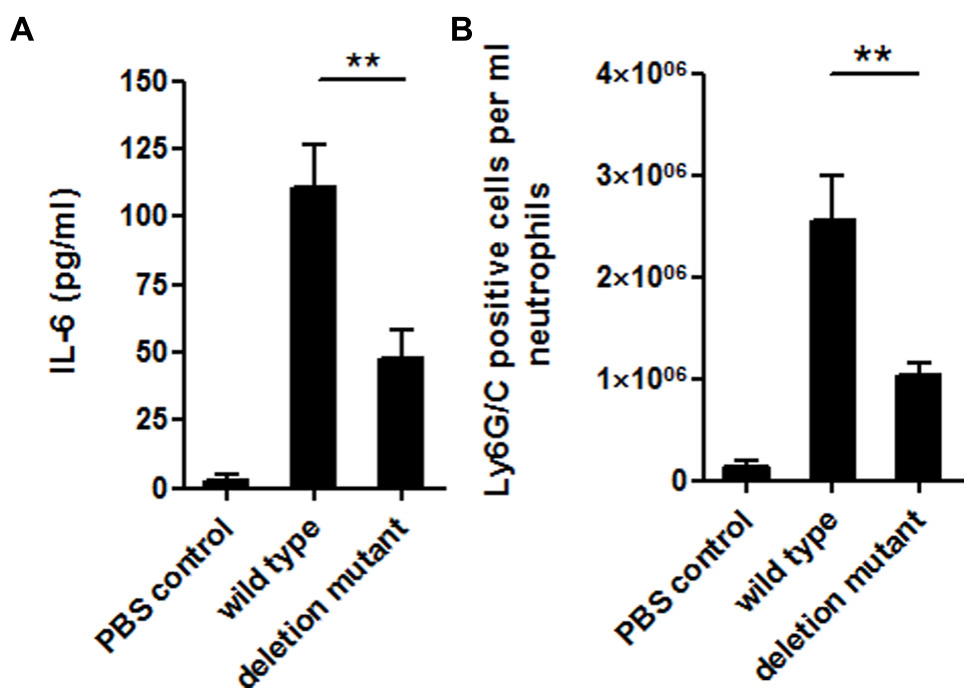
**Host inflammatory response.** Levels of IL-6 **(A)** and number of infiltrating Ly6G/C positive cells **(B)** in the peritoneal cavity of BALB/c mice after intraperitoneal infection with *E. faecalis* OG1RF wild-type and OG1RFΔ*mafR* mutant strain. PBS treated animals served as negative controls. Each bar represents the mean ± SD of three independent experiments, ∗∗*p* < 0.05.

To investigate the influence of the MafR regulator *in vivo*, a peritonitis infection model was used comparing the *mafR* deletion mutant strain (OG1RFΔ*mafR*) and the corresponding isogenic wild-type strain (OG1RF). Our results suggest a role of MafR in bacterial virulence. A significant higher degree of inflammation could be obtained in the peritoneal cavity of mice infected with the wild-type bacteria compared to the *mafR* deletion mutant. This finding was indicated on the cytokine level by a higher release of IL-6 (**Figure 7A**), an important systemic inflammatory cytokine, and on the cellular basis by higher numbers of infiltrating neutrophils (**Figure 7B**) to the site of infection. It is important to state that even though the number of infiltrating neutrophils was 2.5 times higher if mice were infected with the wild-type bacteria compared to the mutant strain, no significant reduction in the bacterial loads within the peritoneal cavity could be observed (data not shown); this also highlights the possible role of the MafR regulator in bacterial virulence. Taken together, our observations clearly suggest an *in vivo* role of the MafR regulator in the course of infections caused by *E. faecalis*. The precise underlying molecular mechanisms of how this regulatory axis is contributing to bacterial virulence, however, need to be elucidated in further studies.

## Conclusion

Our understanding of the mechanisms involved in the pathogenicity of *E. faecalis* is still very limited. In this work, we have constructed a deletion mutant strain to investigate the function of the *mafR* gene. By genome-wide microarrays, quantitative RT-PCR assays, and complementation studies, we have shown that MafR increases the expression of numerous genes. The most significant target genes are involved in the utilization of carbon sources. Associated to this fact, we have found that the growth of the *mafR* deletion mutant strain is impaired in media containing glycerol, maltose, or mannitol. In addition, we have shown that enterococcal cells deficient in MafR induce a more moderate inflammatory response in a mouse peritonitis model. We propose that MafR facilitates the adaptation of *E. faecalis* to particular host niches and, consequently, it contributes to its potential virulence.

## Author Contributions

SR-C, ME, OG, and AB designed the study. SR-C and OG performed laboratory work. SR-C, ME, OG, and AB performed data analysis and wrote the manuscript. All authors read and approved the final manuscript.

## Conflict of Interest Statement

The authors declare that the research was conducted in the absence of any commercial or financial relationships that could be construed as a potential conflict of interest.

## Footnotes

1 www.bioconductor.org

2 https://www.ncbi.nlm.nih.gov/geo/query/acc.cgi?acc=GSE75409

3 http://hydra.icgeb.trieste.it/dna/bend_it.html

## Abbreviations

ABC transporter: ATP-binding cassette transporter
BHI: Brain Heart Infusion
M9YE: M9 minimal medium supplemented with yeast extract
OD: optical density
PRD: PTS regulation domain
PTS: phosphoenolpyruvate:carbohydrate phosphotransferase system
