# Supplementary material for: Global Regulation of Gene Expression by the MafR Protein of Enterococcus faecalis
Source: Front Microbiol. 2016 Jan 11;6:1521. doi: 10.3389/fmicb.2015.01521 (PMC4707282; doi:10.3389/fmicb.2015.01521)
Supplement: Supplementary file 1 [file Table_1.DOC]

**TABLE S1.** Genes differentially expressed in MafR-lacking cells

| **Locus tag**  OG1RF_10064  OG1RF_10107  OG1RF_10108  OG1RF_10198  OG1RF_10296  OG1RF_10297  OG1RF_10298  OG1RF_10433  OG1RF_10434  OG1RF_10682  OG1RF_10683  OG1RF_10684  OG1RF_10746  OG1RF_10747  OG1RF_10748  OG1RF_10749  OG1RF_10979  OG1RF_11004  OG1RF_11005  OG1RF_11008  OG1RF_11009  OG1RF_11133  OG1RF_11134  OG1RF_11135  OG1RF_11136  OG1RF_11137  OG1RF_11138  OG1RF_11146  OG1RF_11147  OG1RF_11148  OG1RF_11149  OG1RF_11150  OG1RF_11184  OG1RF_11185  OG1RF_11186  OG1RF_11584  OG1RF_11585  OG1RF_11590  OG1RF_11591  OG1RF_11592  OG1RF_11611  OG1RF_11612  OG1RF_11613  OG1RF_11614  OG1RF_11615  OG1RF_11616  OG1RF_11753  OG1RF_11761  OG1RF_11762  OG1RF_11763  OG1RF_11775  OG1RF_11940  OG1RF_11944  OG1RF_11948  OG1RF_11949  OG1RF_11950  OG1RF_11951  OG1RF_11956  OG1RF_11957  OG1RF_11958  OG1RF_11959  OG1RF_12167  OG1RF_12249  OG1RF_12303  OG1RF_12397  OG1RF_12398  OG1RF_12399  OG1RF_12400  OG1RF_12401  OG1RF_12402  OG1RF_12403  OG1RF_12404  OG1RF_12405  OG1RF_12425  OG1RF_12426  OG1RF_12561  OG1RF_12562  OG1RF_12563  OG1RF_12564  OG1RF_12565  OG1RF_12566  OG1RF_12567  OG1RF_12568  OG1RF_12569  OG1RF_12570  OG1RF_12571  OG1RF_12572 | **V583**  EF0071  EF0114  EF0115  EF0253  EF0411  EF0412  EF0413  EF0695  EF0696  EF0956  EF0957  EF0958  EF1013  EF1014  EF1015  EF1016  EF1207  EF1233  EF1234  EF1237  EF1238  EF1343  EF1344  EF1345  EF1347  EF1348  EF1349  EF1358  EF1359  EF1360  EF1361  EF1362  EF1395  EF1396  EF1397  EF1920  EF1921  EF1927  EF1928  EF1929  EF1950  EF1951  EF1952  EF1953  EF2213  EF2221  EF2222  EF2223  EF2235  EF2559  EF2563  EF2567  EF2568  EF2569  EF2570  EF2575  EF2577  EF2578  EF2579  EF2863  EF2966  EF3023  EF3134  EF3135  EF3136  EF3137  EF3138  EF3139  EF3140  EF3141  EF3142  EF3157  EF3158  EF3316  EF3317  EF3318  EF3319  EF3320  EF3321  EF3322  EF3323  EF3324  EF3325  EF3326  EF3327 | **Gene**  *aldA*  *mtlA2*  *mltF2*  *mtlD*  *lacD1*  *pgmB*  *map*  *exp5*  *bglX*  *nplT*  *dexB*  *malL*  *gldA*  *dhaK*  *moaB*  *modA*  *glpF2*  *glpO*  *glpK*  *ulaB*  *treB*  *nifJ2*  *selD*  *ygfJ*  *arcC3*  *ygeW*  *ygeY*  *dpaL*  *endOF3*  *eda*  *uxuA*  *sorA*  *sorB2*  *sorD*  *sorC*  *gldA2*  *gnd2*  *trePP*  *yvdM*  *mdh2*  *oadA*  *citX*  *citF*  *citE*  *citD*  *citC*  *gcdB* | **Log_2_FC**  -4.68  -4.52  -3.89  -3.52  -6.40  -6.40  -6.37  -3.67  -3.44  -3.98  -4.00  -4.11  -4.50  -3.70  -4.09  -4.06  -3.37  -3.61  -3.72  -3.17  -3.46  -4.20  -4.20  -4.48  -3.73  -4.11  -4.25  -4.92  -5.04  -5.01  -4.95  -3.20  -3.30  -3.10  -3.70  -3.43  -3.47  -3.26  -3.31  -3.32  -3.84  -4.03  -3.34  -4.81  -3.66  -4.14  -5.09  -4.98  -5.39  -6.05  -3.28  -3.12  -3.03  -3.53  -3.15  -3.12  -3.13  -3.21  -3.39  -3.88  -3.21  -4.43  -3.17  -4.49  -4.17  -4.34  -4.6  -4.64  -4.87  -4.35  -3.94  -3.97  -3.73  -4.07  -3.97  -3.07  -3.6  -3.86  -3.98  -4.19  -4.30  -4.39  -3.06  -4.68  -4.91  -5.38  -4.66 | **Description**  glycoside hydrolase  family 20 glycosyl hydrolase  endoribonuclease L-PSP  aldehyde dehydrogenase  PTS family mannitol porter, EIICB component  PTS family fructose/mannitol porter component IIA  mannitol-1-phosphate 5-dehydrogenase  PTS family fructose/mannitol (fru) porter component IIA  tagatose-bisphosphate aldolase  beta-phosphoglucomutase  family 65 glycosyl hydrolase  PTS family porter component IIABC  PTS family lactose/cellobiose porter component IIC  hypothetical protein  hypothetical protein  hypothetical protein  CCS family citrate carrier protein  ABC superfamily ATP binding cassette transporter, membrane protein  ABC superfamily ATP binding cassette transporter, substrate-binding protein  endonuclease/exonuclease/phosphatase  putative beta-glucosidase  sugar ABC transporter ATP-binding protein  sugar ABC transporter ATP-binding protein  sugar ABC superfamily ATP binding cassette transporter, sugar-binding protein  neopullulanase  glucan 1,6-alpha-glucosidase  oligo-1,6-glucosidase  glycerol dehydrogenase  glycerone kinase PTS family porter component IIA  dihydroxyacetone kinase  dihydroxyacetone kinase  hypothetical protein  molybdenum cofactor biosynthesis protein B  molybdopterin-binding domain protein  molybdenum ABC superfamily ATP binding cassette transporter, binding protein  C4-dicarboxylate anaerobic carrier  putative ribosylpyrimidine nucleosidase  MIP family glycerol uptake facilitator protein GlpF  glycerol-3-phosphate oxidase  glycerol kinase  phosphosugar isomerase  phosphosugar-binding protein  PTS system mannose/fructose/sorbose transporter subunit IID  PTS family mannose/fructose/sorbose porter component IIC  PTS family ascorbate porter, IIB component  PTS system mannose/fructose/sorbose transporter subunit IIA  PTS family trehalose porter, IIBC component  ABC superfamily ATP binding cassette transporter, binding protein  carbohydrate ABC superfamily ATP binding cassette transporter, membrane protein  ABC superfamily ATP binding cassette transporter, membrane protein  glucuronyl hydrolase  pyruvate:ferredoxin oxidoreductase  YqeB family selenium-dependent molybdenum hydroxylase system protein  selenide, water dikinase  putative cysteine desulfurase  YgfJ family molybdenum hydroxylase accessory protein  selenium-dependent molybdenum hydroxylase 1  carbamate kinase  carbamoyltransferase YgeW  M20/DapE family protein YgeY  diaminopropionate ammonia-lyase  mannosyl-glycoprotein endo-beta-N-acetylglucosaminidase  BglG family transcriptional antiterminator  family 8 polysaccharide lyase  2-dehydro-3-deoxyphosphogluconate aldolase  mannonate dehydratase  PTS system mannose/fructose/sorbose transporter subunit IIA  PTS family mannose/fructose/sorbose porter, IIB component  PTS system mannose/fructose/sorbose transporter subunit IID  PTS family mannose/fructose/sorbose porter component IIC  putative glycerol dehydrogenase  D-isomer specific 2-hydroxyacid dehydrogenase  6-phosphogluconate dehydrogenase  glycosyl hydrolase  beta-phosphoglucomutase  malate dehydrogenase (oxaloacetate-decarboxylating) (NADP(+))  oxaloacetate decarboxylase  2-(5''-triphosphoribosyl)-3'-dephosphocoenzyme-A synthase  citrate (pro-3S)-lyase  citrate (Pro-3S)-lyase  citrate lyase acyl carrier protein  [citrate [pro-3S]-lyase] ligase  hypothetical protein  glutaconyl-CoA decarboxylase  sodium ion-translocating decarboxylase  hypothetical protein  citrate transporter | **KEGG pathways**  ND  Other glycan degradation  Amino sugar and nucleotide sugar metabolism  Metabolic pathways  Biosynthesis of secondary metabolites  ND  ND  Fructose and mannose metabolism  Phosphotransferase system (PTS)  Fructose and mannose metabolism  Phosphotransferase system (PTS)  Fructose and mannose metabolism  Fructose and mannose metabolism  Metabolic pathways  Microbial metabolism in diverse environments  Phosphotransferase system (PTS)  Galactose metabolism  Metabolic pathways  Starch and sucrose metabolism  Starch and sucrose metabolism  Metabolic pathways  Glycolysis/Gluconeogenesis  Starch and sucrose metabolism  Amino sugar and nucleotide sugar metabolism  Phosphotransferase system (PTS)  Phosphotransferase system (PTS)  ND  ND  ND  ND  ABC transporters  ABC transporters  ND  Cyanoamino acid metabolism  Starch and sucrose metabolism  Metabolic pathways  Biosynthesis of secondary metabolites  ABC transporters  ABC transporters  ABC transporters  ND  ND  Galactose metabolism  Starch and sucrose metabolism  Metabolic pathways  Glycerolipid metabolism  Metabolic pathways  ND  Glycerolipid metabolism  Metabolic pathways  Glycerolipid metabolism  Metabolic pathways  ND  ND  ND  ABC transporters  ND  ND  ND  ND  Glycerolipid metabolism  Metabolic pathways  ND  ND  Fructose and mannose metabolism  Amino sugar and nucleotide sugar metabolism  Metabolic pathways  Phosphotransferase system (PTS)  Fructose and mannose metabolism  Amino sugar and nucleotide sugar metabolism  Metabolic pathways  Phosphotransferase system (PTS)  Fructose and mannose metabolism  Amino sugar and nucleotide sugar metabolism  Metabolic pathways  Phosphotransferase system (PTS)  Fructose and mannose metabolism  Amino sugar and nucleotide sugar metabolism  Metabolic pathways  Phosphotransferase system (PTS)  Starch and sucrose metabolism  Phosphotransferase system (PTS)  ABC transporters  ABC transporters  ABC transporters  ND  Metabolic pathways  Microbial metabolism in diverse environments  Carbon metabolism  ND  Selenocompound metabolism  Metabolic pathways  ND  ND  ND  Purine metabolism  Arginine and proline metabolism  Nitrogen metabolism  Microbial metabolism in diverse environments  Carbon metabolism  ND  ND  ND  ND  ND  ND  Pentose phosphate pathway  Glyoxylate and dicarboxylate metabolism  Metabolic pathways  Microbial metabolism in diverse environments  Carbon metabolism  Pentose and glucuronate interconversions  Metabolic pathways  Fructose and mannose metabolism  Amino sugar and nucleotide sugar metabolism  Metabolic pathways  Phosphotransferase system (PTS)  Fructose and mannose metabolism  Amino sugar and nucleotide sugar metabolism  Metabolic pathways  Phosphotransferase system (PTS)  Fructose and mannose metabolism  Amino sugar and nucleotide sugar metabolism  Metabolic pathways  Phosphotransferase system (PTS)  Fructose and mannose metabolism  Amino sugar and nucleotide sugar metabolism  Metabolic pathways  Phosphotransferase system (PTS)  ND  ND  Pentose phosphate pathway  Glutathione metabolism  Metabolic pathways  Biosynthesis of secondary metabolites  Microbial metabolism in diverse environments  Carbon metabolism  ND  Starch and sucrose metabolism  Pyruvate metabolism  Carbon metabolism  Two-component system  Pyruvate metabolism  Metabolic pathways  Two-component system  Two-component system  Two-component system  Two-component system  Two-component system  ND  Pyruvate metabolism  Metabolic pathways  ND  ND  ND |
| --- | --- | --- | --- | --- | --- |

ND: no determined
